# Continuous renal replacement therapy combined with double filtration plasmapheresis in the treatment of severe lupus complicated by serious bacterial infections in children: A case report

**DOI:** 10.1515/biol-2022-0477

**Published:** 2022-09-19

**Authors:** Lei Zhang, Feng Wei, Guo-Yun Su, Bo Lin, Wei-Guo Yang

**Affiliations:** Department of Pediatric Intensive Care Unit, Shenzhen Children’s Hospital, No. 7019 Yitian Road, Futian District, Shenzhen, 518000, Guangdong Province, China

**Keywords:** continuous renal replacement therapy, double filtration plasmapheresis, systemic lupus erythematosus, sepsis, multiple organ dysfunction syndrome

## Abstract

There are few reports available on the combination therapy of continuous renal replacement therapy (CRRT) and double filtration plasmapheresis (DFPP) in patients with systemic lupus erythematosus (SLE) complicated by severe bacterial infections, especially in children. A 14-year-old female child with recurrent SLE complicated by severe sepsis-induced multiple organ dysfunction syndrome was administered CRRT combined with DFPP for blood purification in addition to routine immunosuppressant therapy. The changes in autoantibodies, cytokines, and coagulation function indexes of the patient before and after treatment were compared to explore the effect of such therapy on progression and prognosis. After DFPP therapy, significant decreases in the levels of double-stranded DNA antibody, cytokines interleukin (IL)-6, IL-10, and procalcitonin (PCT) were observed. Fibrinogen (Fib) decreased and needed to be replenished following DFPP. After CRRT combined with DFPP, the patient began to urinate sparingly (urine volume was < 50 mL/day) at the seventh week, the urine volume was > 400 mL/day (up to 560 mL/day) at the ninth week (63 days), and the urine volume was >1,000 mL/day at the tenth week, at which time the renal function had fully recovered. DFPP may reduce the plasma Fib concentration, which needs to be replenished in a timely manner. CRRT combined with DFPP shows efficacy in patients with SLE, but the coagulation function requires close monitoring.

## 1 Introduction

Systemic lupus erythematosus (SLE) is a common autoimmune disease. Juvenile SLE is a rare disease, with an incidence of 0.3–0.9/100,000 children per year and a prevalence of 3.3–8.8/100,000 children [1]. The fatality rate of adults with severe SLE who are admitted to the intensive care unit (ICU) due to organ failure can reach 30–60%. Studies at home and abroad show that SLE complicated by a severe infection is the most common cause of death in patients [2,3,4].

This case presents a child with recurrent SLE complicated by a serious bacterial infection. After continuous renal replacement therapy (CRRT) combined with double filtration plasmapheresis (DFPP) for blood purification, the concentrations of autoantibodies and cytokines were rapidly reduced, and the organ function injury was alleviated in the patient; the function of her organs recovered completely. All data including clinical course and laboratory findings were collected from the patient’s records in the hospital. Inflammatory cytokines including interleukin (IL)-6, IL-10, and procalcitonin (PCT) were dosed in a single serum sample by using flow cytometry (HA-330 disposable perfusion cartridge, Jafron Biomedical, Zhuhai, China). Autoantibody was measured by a fast test kit using the immunofluorescence assay method on a Getein 1600 device (Getein Biotech, Nanjing, China).

### 1.1 Case data

A 14-year-old female patient was admitted to the hospital on September 23, 2021, complaining of “edema for more than 3 weeks, aggravated with labored breathing for 3 days.” The patient had suffered from edema 3 weeks before admission and had been treated with acupuncture. Three days prior, the edema in her lower limbs had been significantly aggravated, with cyanosis of the skin at her right lower limb, accompanied by oliguria and labored breathing. There was no fever, cough, gross hematuria, and rash, but she did experience loose stools during the course of the disease. In the physical examination, a temperature of 37.2℃, a pulse rate of 106 beats/min, a respiratory rate of 45 breaths/min, a blood pressure reading of 86/56 mmHg, a mask oxygen level of 98%, and a weight of 55 kg were recorded. The patient exhibited a blurred mind, intermittent restlessness, a grimace when in pain, forced sitting, a pale complexion, and a swollen face, trunk, and limbs, especially the lower limbs; she also had purplish-red tissues around her right knee, with tenderness and a visible seepage on the surface. Her bilateral pupils were equally large and sensitive to light reflection, and her lips were pale with a flaring of the nares. The patient had a soft neck and experienced shortness of breath, but there were no obvious three depression signs. Fine rales were heard from the bases of both lungs. The patient was found to have a strong cardiac sound and a heart rate of 106 beats/min, with a regular heart rhythm and no murmur. Additionally, abdominal distension, abdominal wall swelling, unclear palpation of the liver and spleen, positive shifting dullness, normal muscle force and muscular tone, obvious swelling of the lower limbs, cold acra, no pattern, weakened pulses of the dorsalis pedis artery, and a clot retraction time of 3 s were observed.

The patient’s past medical history revealed that 6 years prior, she had been diagnosed with SLE, lupus nephritis (LN), and lupus pneumonia. After steroid and cyclophosphamide pulse therapy, oral prednisone, hydroxychloroquine, and other regular therapies were administered, showing stable conditions. Six months prior, she discontinued all drugs due to a fear of possible side effects.

The blood gas values from the laboratory examination after admission were as follows: a potential of hydrogen (pH) of 7.44, partial pressure of oxygen of 197 mmHg, partial pressure of carbon dioxide of 17 mmHg, Na of 97 mmol/L, K of 6.2 mmol/L, Carbonic acid hydrogen radical of 15.3 mmol/L, base excess of 11.9, ionized calcium of 0.97 mmol/L, and a glucose of 4.88 mmol/L.

Results from the blood routine examination were as follows: white blood cells (WBCs) of 7.01 × 10^9^/L, neutrophils (NEUT) of 6.23 × 10^9^/L, red blood cells (RBCs) of 2.19 × 10^12^/L, hemoglobin of 66 g/L, platelet of 131 × 10^9^/L, and a C-reactive protein of 79.18 mg/L.

The coagulation function showed an activated partial thromboplastin time (APTT) of 47 s, prothrombin time (PT) of 18.5 s, thrombin time (TT) of 16.6 s, international normalized ratio of 1.66, D–Dimer of 7.40 μg/mL, and fibrinogen degradation product (FDP) of 22.4 μg/mL.

Hepatic and renal functions and myocardial enzyme results showed a total protein of 53.2 g/L, albumin (ALB) of 21.4 g/L, alanine aminotrans of 144 IU/L, aspartate transaminase of 216 IU/L, blood urea nitrogen of 34.31 mmol/L, Serum creatinine of 273.7 μmol/L, creatine kinase of 503.7 IU/L, creatine kinase isoenzyme-MB of 5.5 ng/mL, myoglobin of 1953.2 ng/mL, cardiac troponin I of 0.026 ng/mL, lactic acid of 3.38 mmol/L, and a B-type natriuretic peptide of 1766.3 pg/mL.

The urine routine examination results were as follows: a pH of 5.0, turbid, occult blood (3+), protein (2+), red blood cell of 228.8 p/μL, WBC of 16658.4 p/μL, and a leukocyte esterase 3+.

The autoantibody test results were as follows: anti-double-stranded DNA (ds-DNA) (+), anti-SS-A antibody (SSA) (3+), anti-SS-B antibody (SSB) (3+), anti-Sm antibody (Sm) (−), anti-Jo-1 antibody (−), anti-histone antibody (−), MPO-ANCA (−), PR3-ANCA (−), anti-GBM antibody (−), anti-Sc1-70 antibody (−), anti-nucleosome antibody (−), anti-RNP antibody (−), anti-centromere antibody (−), anti-ribosomal protein antibody (−), and anti-cardiolipin antibody (−).

The bedside echocardiography showed an ejection fraction (EF) of 32%, the myocardial echo was not enhanced, and the left ventricular wall motion was diffusely attenuated.

The blood culture (positive after 9 h) showed a presence of extended-spectrum β-lactamase-negative *Escherichia coli*. The blood culture was negative 1 week later. One month later, the tissue debrided with secretions for culture showed a presence of *Escherichia coli*, *Staphylococcus aureus*, and *Stenotrophomonas maltophilia*.

The admission diagnoses were as follows: severe sepsis (*Escherichia coli*), a soft tissue infection of the right lower limb, SLE (recurrent), multiple organ dysfunction syndrome (MODS) (shock, acute kidney injury, heart failure, and disseminated intravascular coagulation [DIC]), and an electrolyte disturbance (hyposodium, hyperpotassium, hypocalcium, and metabolic acidosis).


**Informed consent:** Informed consent has been obtained from all individuals included in this study.
**Ethical approval:** The research related to human use has been complied with all the relevant national regulations, institutional policies and in accordance with the tenets of the Helsinki Declaration, and has been approved by the authors’ institutional review board or equivalent committee.

### 1.2 Treatment regimen

1. The anti-infection treatment comprised meropenem (40 mg/kg, every 12 hours (q12h)) for three consecutive weeks and then ceftazidime (50 mg/kg, q12h) for two consecutive weeks, followed by levofloxacin (10 mg/kg, one a day (qd)) for three consecutive weeks [5].
2. The antishock treatment consisted of the appropriate amount of liquid resuscitation of 500 mL/dayose × 2 doses, 20–30 min of infusion, and an infusion of ALB and leukocyte-reduced red blood cells. Epinephrine (0.1–1 μg/kg/min) and norepinephrine (0.1–1 μg/kg/min) were administered for maintenance.
3. The immunosuppressant treatment was initially administered with small doses of methylprednisolone intravenous drip (40 mg/dose, q12h) for two consecutive weeks, prednisone tablets (30 mg, twice a day (bid)) for four consecutive weeks, prednisone tablets (60 mg, qd) for a week, and prednisone tablets (50 mg, qd) for three consecutive weeks, then reduced to 40 mg qd (1 mg/kg, qd) around the third month. Intravenous immunoglobulin (IVIG) at 400 mg/kg was administered for 5 days, and hydroxychloroquine (0.2, qd) was administered after 1 month and then decreased gradually.
4. Blood purification required CRRT as early as possible and replacement fluid adjustments in Na^+^ and K^+^ concentrations in real time to keep a slow rise in Na^+^ and prompt the correction of hypokalemia and acidosis. The patient was administered 5 DFPP therapies within 1 week after admission (qd × 3, every other day (qod) × 2).
  For the CRRT, a double-lumen catheter was indwelt in the right subclavian vein for vascular access (Asahi Kasei PlasautoΣ AEF 07 filter, blood flow velocity of 100–200 mL/min, waste dosage of 35–55 mL/kg/h, replacement fluid:dialysis fluid = 1:1). At the beginning, the filter should be replaced every 12 h, and after clinical improvement and decrease in cytokine concentration in the patients, the use time of filter should be prolonged (up to 70 h). Heparin was used for anticoagulation in the first 3 days, and the activated clotting time (ACT) of whole blood was monitored every 2 h for control at 160–180 s after filtration. The blood routine examination and coagulation function were monitored daily. In case of platelets <10 × 10^9^/L, homotype platelets were transfused, and in case of antithrombin III < 50%, the homotype plasma was transfused. Three days later, her shock was corrected, but she continued to have a low platelet count. Citric acid was then administered for local anticoagulation. The *in vitro* ionized calcium (iCa) was maintained at 0.25–0.4 mmol/L, and the *in vivo* iCa was >0.9 mmol/L. Additionally, low molecular weight heparin calcium was injected subcutaneously to prevent lower extremity thrombosis.
  For the DFPP therapy, a double-lumen catheter was indwelt at the right subclavian vein for vascular access, and an OP05W plasma separator was used as a primary filter and the EC30W as a secondary filter (Asahi Kasei, Japan). Heparin was used for anticoagulation, and the ACT was controlled at 180–200 s. Under extracorporeal circulation, the whole blood flowed through a primary filter (80–100 mL/min), with a separation/blood flow of 20%. The filtered plasma was filtered again through a secondary filter, and then transfused back into the patient. The intercepted plasma fraction flowed into the liquid waste bag to stop the plasma separation. The secondary membrane was rinsed using 500 mL of 5% ALB to further filter the retained plasma fractions in the secondary filter, and the residual plasma fractions in the secondary filter were discarded. The plasma volume per treatment was twice the plasma volume, and 20 g ALB was used each time. After DFPP, 200 mL of fresh frozen plasma or 1 g of human Fib was transfused based on the coagulation function.
5. Debridement and skin grafting were performed at the seventh week, comprising five debridements and two skin grafts.

### 1.3 Clinical efficacy

The patient’s hyperkalemia was corrected after CRRT for 3 h, and the blood sodium rose steadily to 130 mmol/L after approximately 3 days. Once her blood pressure had gradually stabilized, all vasoactive drugs were discontinued; her cardiac EF value recovered to 44% within 2 days and 52% within a month. Approximately 2 weeks later, the patient’s platelet count was maintained above 20 × 10^9^/L, and the creatinine had decreased to 65.3 μmol/L (normal range is 33–75 μmol/L) within 3 days. Allowing for continuous anuria, CRRT was continued to keep the creatinine within the normal range; the patient began to urinate sparingly (urine volume was< 50 mL/day) at the seventh week, the urine volume was >400 mL/day (up to 560 mL/day) at the ninth week (63 days), and the urine volume gradually increased to >1,000 mL/day at the tenth week, at which time the renal function had fully recovered.

After DFPP, the level of ds-DNA antibody had decreased significantly, but SSA and SSB antibody levels had not changed significantly; complement C3 (C3) had significantly improved, but the remaining levels had not changed significantly. The levels of cytokines interleukin-6 (IL-6), interleukin-10 (IL-10), and PCT had significantly decreased, as shown in Tables 1 and 2.

**Table 1:** Changes in antibody levels before and after DFPP

Date	ds-DNA IU/mL	SSA	SSB	Immunoglobulin G (g/L)	Immunoglobulin A (g/L)	Immunoglobulin M (g/L)	C3 (g/L)	Complement C4 (g/L)
Before treatment	302	3+	3+	16.52	3.26	0.56	0.20	0.02
3 DFPPs	113	3+	3+	—	—	—	—	—
5 DFPPs	30	3+	3+	18.41	2.13	0.58	0.49	0.03

**Table 2:** Changes in inflammatory factors before and after DFPP

Date	Interleukin-2 (pg/mL)	Interleukin-4 (pg/mL)	IL-6 (pg/mL)	IL-10 (pg/mL)	Tumor necrosis factor-α (pg/mL)	Interferon-gamma (pg/mL)	Interleukin-17A (pg/mL)	PCT (ng/mL)
Before treatment	0.47	2.20	10209.7	23.79	3.72	0.9	1.10	52.20
3 DFPPs	0.33	1.55	15.19	6.34	2.17	1.10	0.31	4.36
5 DFPPs	0.88	3.24	7.58	2.87	3.42	0.8	1.22	

After DFPP, the APTT, PT, and TT were prolonged, which was related to heparin anticoagulation, while Fib, D–Dimer and FDP decreased significantly, as shown in Table 3.

**Table 3:** Changes in coagulation functions before and after DFPP

Item	1st DFPP (before/after)	2nd DFPP (before/after)	3rd DFPP (before/after)	4th DFPP (before/after)	5th DFPP (before/after)
APTT (s)	44.3/76.1	52.3/89.7	62.9/89.2	60.8/110.8	40.6/52.8
PT (s)	16.5/20.3	17.3/23.1	18.3/26.5	17.4/24.8	15.5/20.4
TT (s)	13.7/18.9	17.4/28.0	22.7/30.7	24.9/36.9	17.6/23.4
Fib (g/L)	3.14/1.41	1.76/0.72	1.18/0.60	1.22/0.45	1.31/0.60
AT-III (%)	54/59	73/59	66/66	69/77	81/69
D–D (μg/mL)	7.34/5.77	7.48/3.75	4.44/2.65	2.88/2.29	2.79/2.08
FDP (μg/mL)	25.59/21.59	25.29/15.86	14.17/7.57	11.16/8.60	10.38/6.59

**Figure 1:**
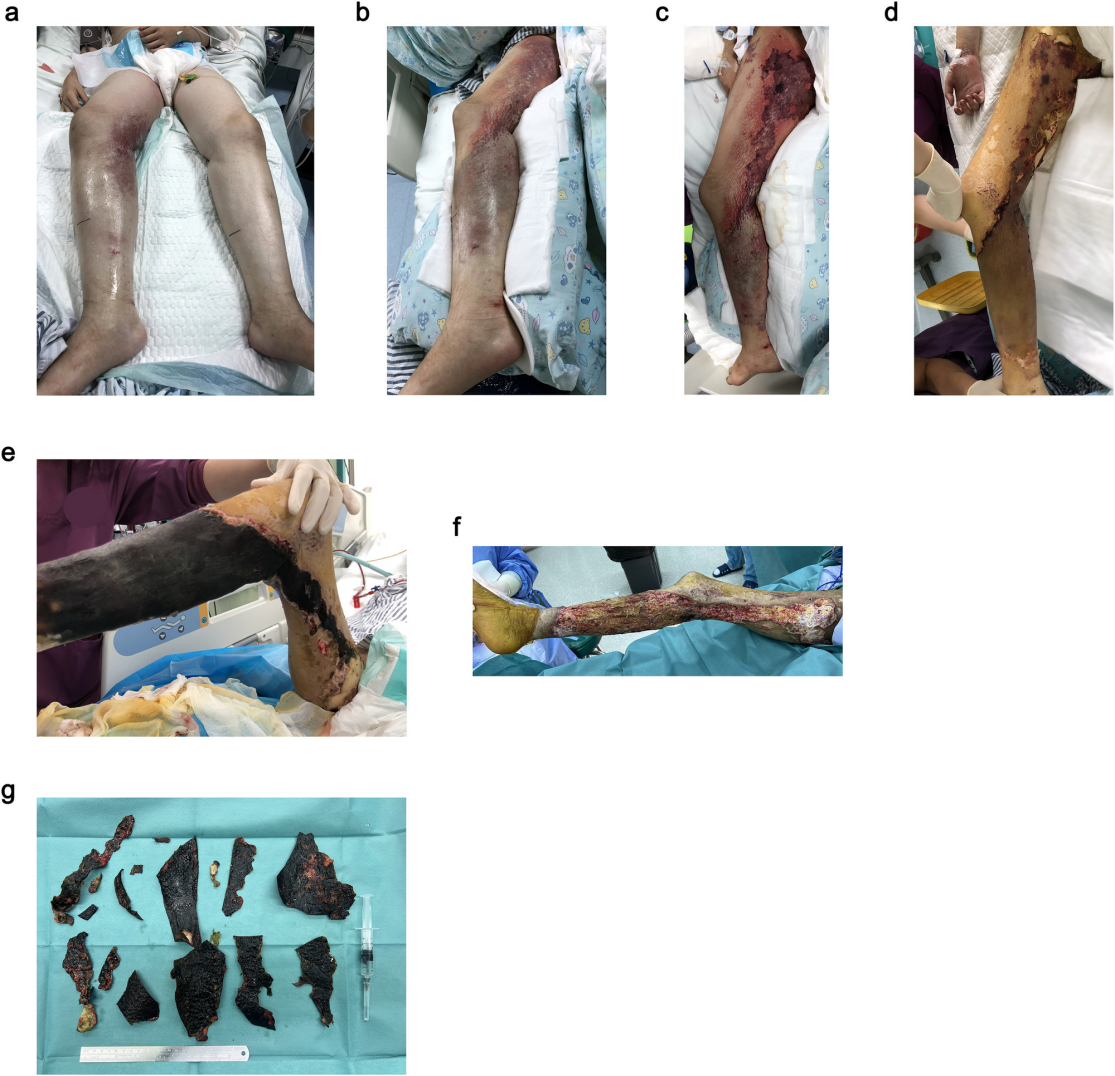
Soft tissue infection in the patient’s right lower limb. (a) Edema at both lower extremities, with stasis at the right side. (b) Edema alleviated, with a clear boundary between the infected areas and normal tissues. (c) Necrotizing ecchymoses at the infection site of the right lower limb. (d) Gradually drying infection site of the right lower limb. (e) Necrotizing eschars of skin at the infection site with clear boundaries. (f) Skin and subcutaneous tissue necrosis, partial tendons involved, superficial venous embolism found in the surgery. (g) Necrotic skin and tendons cut off during debridement.

After the CRRT and DFPP therapy, the tissue edema was relieved and had gradually dried, forming a large area of necrotizing eschars with a clear separation from surrounding tissues. After five debridements and two skin grafts, her right lower limb was gradually recovering without disability (Figure 1).

## 2 Discussion

The patient had suffered from an SLE relapse complicated by a serious bacterial infection of the skin and soft tissues, which then progressed to MODS (shock, acute kidney injury, heart failure, and DIC). In addition to the impaired organ function caused by the inflammatory activity of SLE itself, the release of inflammatory factors induced by sepsis may lead to MODS. It is quite difficult to distinguish whether the final adverse outcome is dominated by the infection or organ failure due to SLE activity.

The value of CRRT in the treatment of severe sepsis has been previously recognized [5,6]. Its primary functions are to clear small and medium molecules and control fluid balance, but it is somewhat limited in the removal of macromolecular substances produced by SLE. Therefore, CRRT must be combined with therapeutic exchange plasmapheresis (TPE). It has been reported that TPE is performed every other day for at least 7–14 therapies [7]. The 2019 guidelines of the American Society for Apheresis recommend plasma exchange for patients with SLE accompanied by severe complications [8]. Double filtration plasmapheresis can selectively remove macromolecular substances in plasma and reduce the plasma dosage, and it has become an effective alternative to TPE therapy in recent years. It has been reported that in adult patients, DFPP can effectively remove autoantibodies and improve the long-term renal survival rate in patients with LN accompanied by thrombotic microangiopathies [9,10,11], but severely infected patients were excluded in all included ones, and DFPP therapy was rarely seen in children.

In this case, the patient was administered DFPP for a total of five courses. After three courses, ds-DNA had decreased significantly, and after five courses, it had reached the normal level; C3 rose, which is consistent with the report [11]. Its clearance efficiency was much higher than that of conventional TPE. However, SSA and SSB remained at high levels, which might be ascribed to the patient’s severe skin lesions. In theory, DFPP therapy would decrease the IgG level, but the patient had been treated with IVIG before, which might have affected the outcome. Cytokines play a major role in the pathophysiology of sepsis. The addition of IL-6, IL-8, and PCT to quick sepsis-related organ failure assessment scores improved the accuracy of early MOD prediction. Increased levels of pro-inflammatory cytokines in the serum result in a disturbance in the immune system, inducing multi-organ failure that may cause elevated mortality rates among ICU patients [12,13]. The current study showed that the progression of sepsis in ICU patients may be prevented with cytokine hemadsorption applied as an immunomodulator therapy [14]. It is worth noting that inflammatory factors IL-6, IL-10, and PCT in the patient were abnormally elevated before DFPP, and after three courses of DFPP, IL-6 and IL-10 returned to normal levels, while PCT decreased by ten times. It has been suggested that DFPP has a good clearance effect on cytokines caused by severe infection, thus it is beneficial in reducing the organ functional lesion.

Double filtration plasmapheresis can clear clotting factors in plasma. Table 3 shows that Fib decreased significantly after DFPP, with a replenished plasma or human Fib. During the course of DFPP, the patient’s platelet count was still at a low level (10–20 × 10^9^/L), and 200 mL of plasma or 1 g human Fib was transfused within a timely manner after each treatment, without resulting in hemorrhage and other adverse events. After that DFPP, D–Dimer, and FDP decreased significantly, which may have positive implications for thrombotic diseases.

The patient suffered from an SLE relapse and severe organ injury. According to the treatment guidelines [15], hormone therapy or steroid pulse therapy was required. However, allowing for the fact that the patient’s condition was complicated by a serious bacterial infection, hormone therapy might have been detrimental to infection control. If the renal function cannot be recovered for a long time, it will present difficulties in debridement and skin grafting, and it can also affect the survival rate of the skin graft. After admission, the patient was first given CRRT to rapidly correct an electrolyte disturbance and control fluid balance. DFPP was then combined with CRRT to remove autoantibodies and inflammatory factors, reduce organ damage, promote renal function recovery, and provide conditions for subsequent debridement and skin grafting.

## 3 Conclusion

CRRT combined with DFPP can effectively remove inflammatory factors and autoimmune antibodies in the plasma of patients with SLE complicated by severe infections. This is conducive to mitigating the attack of inflammatory factors and autoantibodies to organs and is in favor of organ function recovery. Double filtration plasmapheresis may reduce plasma fibrinogen concentration, which needs to be replenished in a timely manner. CRRT combined with DFPP shows efficacy in patients with SLE, but the coagulation function requires close monitoring.
